# Developing Pretend Play in Autistic Children Using the Playboxes Joint Play Approach as Part of Ongoing Practice

**DOI:** 10.1007/s10803-021-05156-9

**Published:** 2021-07-09

**Authors:** Helen Marwick, Karena Jarvie, Hilary Cowie, Lorna Johnston, Nicola Hammond-Evans, Rachael Cockayne

**Affiliations:** 1 Faculty of Humanities and Social Sciences, School of Education, Lord Hope Building, University of Strathclyde, 141, St James Road, Glasgow, G4 0LT UK; 2 Psychological Services, Children and Families, City of Edinburgh Council, Edinburgh, UK; 3 NHS Lothian, Edinburgh, UK; 4 Additional Support for Learning Services, Children and Families, City of Edinburgh Council, Edinburgh, UK; 5 Present Address: Nottinghamshire Healthcare NHS Trust, Nottingham, UK

**Keywords:** Children, Autism, Joint-play support, Joint attention, Pretend play, Ongoing practice

## Abstract

A repeated measures single subject design was used to examine the effectiveness of a joint play approach embedded in professional practice, in supporting pretend play for autistic children. Seven autistic children, aged 5–8 years, with a placement within a specialist educational provision, and who demonstrated restricted play, participated in weekly sessions using the Playboxes approach over a period of 3 months. Pre- and post-approach pretend play abilities were assessed using the Symbolic Play Test and the Test of Pretend Play. Every child gained increased age-equivalent scores on the Test of Pretend Play, ranging from + 8 to + 30 months. Pretend Play abilities can support developmental outcomes and incorporation of this approach into regular practice could be of value for autistic children.

Autistic children have been reported to show a particular reduced involvement in symbolic play, and limited spontaneous imaginative creativity in pretence (Beyer & Gammeltoft,  2000; Hobson et al., 2013; Janert, 2000; Jarrold et al., 1993; Kasari et al., 2010; Rutherford et al., 2007; Wilson et al., 2017). It is argued that engagement in joint pretend play contributes to the cognitive, social and communicative development of typically developing children (Stern, 1985, 2000; Trevarthen, 2001), and differences in entering into joint imaginative play interactions for autistic children are considered to potentially affect social communication, friendship making and social inclusion (Freeman et al., 2015; O’Connor & Stagnatti, 2011).

Pretend play involves imagining and acting out events, existences, occurrences, activities, and feelings (Fein, 1987; Leslie, 1987; Trevarthen & Marwick, 1986), and can incorporate the symbolic representation of objects and actions, states of being and emotions, as well as the imaginative representation of social roles, relationships and scenarios. Such pretence can embody particular symbolic imaginative representation, such as object substitution, where an object is made to stand for something else (such as a wooden block being made to be a cake), or where an activity, state of being, or emotion is symbolized through expressive action, gesture, sound, body posture or movement (such as a toy horse being made to move slowly because it is ‘tired’). Play with objects involving ‘conventional’ symbolic representation such as making a toy car run, or sitting a doll in a toy highchair, is often referred to as functional play (Jarrold et al., 1993; Lewis et al., 2000; Williams et al., 2001). Pretend play can be seen from around the first year with early functional symbolic representation and object substitution tending to appear before more complex pretence with imagined activities, objects, states, feelings, role play and social scenarios (Thomson & Goldstein, 2019; Westby, 1980). In this way, pretend play activities can be understood to reflect the imaginative representation of conceptual understandings about the world in relation to interactions of people, objects, events and feelings, which, in play, are generated to form a prospectively imagined possible reality (Bruner & Sherwood, 1976; Trevarthen & Marwick, 1986; Winnicott, 1971). These conceptual understandings are argued to be developed through acting upon the world, exploring object affordances, and through co-created meanings constructed in interactions with others, as the ideas, feelings, intentions and perspectives of others are shared and navigated (Bornstein et al., 1996; Emde et al., 1997; Marwick, 2017; Piaget, 1951, 1962; Trevarthen & Marwick, 1986; Van Berckelaer-Onnes, 2003; Vygotsky, 1978). Underpinning these shared understandings are interpersonal processes such as joint attention, cooperative activity, and shared intentionality (Bruner, 1983; Marwick, 2012, 2017; Rutherford et al., 2007; Tomasello et al., 2005; Trevarthen, 2001). In this way, pretend play not only reflects conceptual understandings of the world, but also generates such understandings. Consequently, joint social interactive pretend play in typical development is considered to be an important contributor to interpersonal, conceptual, emotional and communicative development (Bruner & Sherwood, 1976; Goldstein & Lerner, 2018; Marwick & Murray, 2008; Quinn, et al., 2018; Stagnitti et al., 2012; Stern, 1985/2000; Trevarthen, 2001) as well as the development of symbolic representation and imaginative pretence (Bornstein et al., 1996; Emde et al., 1997; Tamis-Lemonda et al., 1998; Trevarthen & Marwick, 1986; Vygotsky, 1978).

Concomitantly, it is argued that the lessened involvement in joint playful engagement reported for autistic children can contribute to altered presentation or development of a range of abilities, including symbolic representation, perspective-taking, joint imaginative play and language development (Hobson et al., 2009; Jordan, 2003; Kasari et al., 2008, 2013; Trevarthen et al., 1998), affecting, in turn, social involvement and interpersonal friendships for the child (Freeman et al., 2015; O’Connor & Stagnitti, 2011). Support for joint attention and engagement in joint playful interactions for young autistic children has consequently been a target of intervention. Studies in which autistic children are supported to become engaged in play activities by, for example, trained professional adult or parent play partners, structured and semi-structured play situations, and peer support, in homes, nurseries, schools and clinical environments, report positive developments not only in joint attention and pretend play abilities (Hobson et al., 2013; Kasari et al., 2006, 2010; Kossyvaki & Papoudi, 2016; Lawton & Kasari, 2012; O’Connor & Stagnitti, 2011; Sherrat, 2002; Wolfberg, 1999; Zercher et al., 2001), but also in language, cognitive abilities and friendships (Chang et al., 2018; Dykstra et al, 2012; Kasari et al., 2012; Stagnitti et al., 2012; Weider & Greenspan, 2003). Such findings can be argued to indicate that the processes underpinning pretend play abilities are able to be supported through engaged interaction, and that lack of demonstration of pretence in play for autistic children does not reflect altered imaginative processes underlying pretence, or an absence of potential (Hobson et al., 2013; Jarrold, 2003; Jarrold et al., 1996; Kasari et al., 2013; Sherratt, 2002).

Nevertheless, while play-based interventions are reported to support playful engaged interactions for young children on the autism spectrum, Kossyvaki and Papoudi (2016) report that the design of the majority of studies in their review of play interventions in schools was found to be less than strong, with, for example, outcome variables lacking precise description. Kasari et al. (2013), similarly reviewing such methodological limitations in studies of children’s play, stress the need for rigorous testing of pretence abilities. Thomson and Goldstein (2019) highlight the variability and range of play behaviours and understandings within pretend play emphasising the need for clarity and precision of description in measuring specific pretense behaviours, and Pierucci et al. (2015) also indicate the need within research for concordant assessment approaches of pretence.

In the current study we aimed to systematically examine the effectiveness of the ‘Playboxes’ joint-play approach to support engagement and pretend play with autistic children, using independent standardized assessments of pretend play both before and after the approach sessions. The Playboxes joint play intervention is a naturalistic approach involving an adult play partner and a child, which uses matched boxes of toys to support engagement and interpersonal play. Previous work on the effectiveness of Playboxes has looked at outcomes using categorised observation within the context of the intervention session, and positive results were found in relation to children’s increased engagement and use of pretence across the sessions. However, we wished to examine the effectiveness of Playboxes on pretend play activities separately from the Playboxes context, in order to minimize the effect of the potential familiarity of co-constructed pretence activities developed within the Playboxes sessions on the imaginative play of the child. This would enable a more rigorous and independent assessment of Playboxes in supporting the pretend play of the children involved in the study and also allow us to examine whether the pretence of the children is generalised to a less conducive context. Additionally, because one of the independent standardized assessments distinguishes between pretend play that is imitated and pretend play which is generated anew by the child, independent generation of pretence by the children would be demonstrated, enabling the indication of evidence of underlying imaginative processes of pretence to be considered. The study would embed the Playboxes approach into ongoing professional practice. If shown to be effective in supporting pretend play for the children involved, this would indicate an accessible support approach, involving short individual training, which could be used by professionals at point of concern, and sustained as needed.

## Methodology

### Design

The Playboxes intervention was carried out over a three-month period as part of the ongoing work of professionals working with autistic children in a range of schools within one education authority. A repeated measures single subject design was used with seven children, with standardised pre-test/post-test measures. Individual case pre- and post-approach play abilities were assessed using the Symbolic Play Test (SPT) (Lowe & Costello, 1988) and the Test of Pretend Play (ToPP, Structured Condition) (Lewis & Boucher, 1997).

### Participants

The study involved seven autistic children aged between 5–8 years. The cohort comprised six boys and one girl; all seven children had an existing diagnosis of autism assessed according to ICD-10 or DSM 1V criteria (APA, 1994; WHO, 1992). Two participants had a concurrent diagnosed moderate learning difficulty (LD). For two participants English was an additional language (EAL). All participants were in the early years of primary education between the first year and the third year of formal schooling.

Six professionals who had been trained in the Playboxes approach were involved in the study: an educational psychologist (EP), three speech and language therapists (SLT), and two teachers; one a teacher in an autism provision within a mainstream school and one a teacher in a specialist provision for children with moderate learning difficulties. The practitioners selected potential participant children from the group of children they were already working with professionally. The selection criteria were: an existing diagnosis of autism, placement within a specialist educational provision, chronological age of between 5 and 8 years at start of intervention, and demonstration of restricted play skills. The seven children were recruited through a direct face-to-face approach to their parents. During the intervention period the participants were not involved in other interventions other than standard practice. Details of the participants and number of sessions are shown in Table 1.

**Table 1:** participant details and number of sessions

Participant	Chronological Age (at start)	Learning Difficulty (LD)/English as an Additional Language (EAL)	Number of Sessions	Professional
1	72 m		10	1
2	71 m		10	1
3	79 m		10	2
4	80 m		10	3
5	74 m	EAL	8	4
6	63 m	LD + EAL	7	5
7	91 m	LD	10	6

### The Playboxes Approach

The ‘Playboxes’ joint-play approach, is informed by intersubjectivity theory (Trevarthen, 2001) and is a play-based method for both the assessment and promotion of active interpersonal engagement, interpersonal communication and shared imaginative representation between a child and an adult interactive partner. The joint-play setting with matched toy-boxes for the child and the adult is designed to facilitate shared interpersonal focus, interpersonal contingency and cooperation, and, in this way, is designed to encourage the child’s motivation to engage with another person and to promote joint imaginative play. Studies of the use of the playboxes approach in weekly sessions of up to 45 min in length, have used categorized observation of play and engagement during the play sessions to assess developments in interpersonal engagement, imaginative play abilities and communication and language abilities for the children involved.

The two matching boxes with lids are decorated according to the particular interests of the child and adult, and each box contains a number of toys, which either directly ‘match’, such as there being identical spinning tops in each box, or are ‘complementary’, such as having train carriages in one box, and train track in the other. Following the typical development of joint play in young children, the toys are selected to encourage ‘expressive-attentive’ ‘joint goal-directed’ and ‘imaginative play’, with: *expressive-attentive* joint play involving both play partners sharing an emotional response to the sound, movement or other expressive properties of a toy, or of each other (such as both enjoying the sound effect of a musical toy); *goal directed* play being where the joint-play has a particular intentional goal, such as rolling a ball to each other, or building up a tower of blocks together; and *imaginative* play (which includes imaginative play with a representational ‘other’, such as a puppet) being where the play involves pretence such as the child pretending to be a dinosaur, or feeding the teddy imagined ice cream with a spoon.

Within the three types of play, and using the matched resources, the adult can: join the child in play, imitate, model, invite and offer comparisons, instigate turn-taking, refer back to shared experiences, offer imaginative scenarios, and give interpersonal effectiveness to the communicative moves and activities of the child, as well as amusing, surprising and intriguing the child. Adults are encouraged to involve the ‘representational other’ toys as an additional participant in the play activities. Playboxes enables both the child and the adult to take the lead in starting or suggesting activities, with each following the other.

### Study Procedure

The practitioners implemented the approach with individual pupils in up to ten weekly one-to-one sessions, which lasted for approximately 40 min. Five practitioners each worked with one child only; the sixth team member worked with two children in separate individual sessions.

The Playboxes sessions generally took place at the same time each week and always with the same practitioner, who was trained in the Playboxes approach. A specially designed Boardmaker© symbol was placed on the child’s visual timetable to aid transition between the classroom and the Playboxes room. The sessions took place in a separate, but familiar and consistent room within the school setting with as few distractions as possible. The child was prepared for the end of each session with a verbal cue, sign, or the use of a timer as appropriate. Sessions varied in length but were typically 30–40 min. On a small number of occasions the sessions were shorter due to unforeseen factors separate from the approach in relation to the child or school setting. Five of the seven children participated in 10 weekly sessions; two children participated in only 7 or 8 sessions due to child or practitioner illness (see Table 1).

### Assessment—Pre and Post Measures

The Symbolic Play Test (SPT) (Lowe & Costello, 1988) and the Test of Pretend Play (ToPP) (Lewis & Boucher, 1997) were carried out before and after the approach period with all participants. All pre-assessments were carried out within the month leading up to the beginning of the approach sessions. Six of the post tests were completed within a month of the last session and one was completed within two months.

The Symbolic Play Test (SPT) (Lowe & Costello, 1988) is a non-verbal measure of conventional symbolic representational early play in children aged 12–36 months. The test records spontaneous non-verbal play activity in a structured situation and does not require expressive speech, or verbal comprehension. Children are sequentially presented with four sets of toys and their spontaneous manipulation of the object is observed and recorded on a standardised checklist. If the child does not spontaneously engage with the toys, neutral prompts such as ‘what can you do with these?’ can be used, following the guidelines. SPT provides age norm equivalents for raw scores equivalent to 13 months and up to 36 months. The test takes about 20 min. All participants in this study were chronologically older than the age range for the test, however, as the children involved in the study showed very limited play, the SPT was considered appropriate to use to provide information about early functional symbolic play abilities.

The Test of Pretend Play (ToPP) (Lewis & Boucher, 1997) is designed for children with a verbal mental age of between 1–6 years, and can be used with children with developmental difficulties in the age range of 1–8 years. It is designed to assess three types of pretend play: substituting one object for another object or person; attributing an imagined property to an object or person; and reference to an absent object, person or substance. In this study, the play was assessed using the ToPP structured conditions procedure. The test involves a number of activities where the adult models an action or instructs the child to carry out an action with, firstly, two functionally related objects, and then progresses to activities requiring the child to substitute one object for another (e.g. a top for a hat), and to attributing an imagined property or reference to an absent object, person or substance. The test comprises four sections: self with everyday objects; toy and non-representational materials; representational toy alone; self alone. The standard scoring procedures yield age equivalent scores for each child within which the generation of novel meanings and ideas receives higher scoring credit than copied meanings and copied symbolic ideas.

Both standardised play assessments were carried out by the practitioners involved in the study, or members of the SLT teams within the participants’ schools. Where possible these were carried out by a different practitioner to the one carrying out the approach for any particular child, however, the practice-based context of this study meant that it was not possible for all participants to have their assessments conducted by practitioners not involved with their sessions. Two of the seven children (Child 3 and Child 7) had their SPT assessments administered by the person carrying out the approach. Three of the seven children (Child 3, Child 4 and Child 7) had their pre and post ToPP assessment administered by the person carrying out the intervention. With the exception of the SPT assessments for Child 2 and Child 5, the pre and post assessments for each child were carried out by the same administrator on each occasion.

## Results

All the children engaged with the tests of pretend play and these were able to be carried out successfully.

**Fig. 1:**
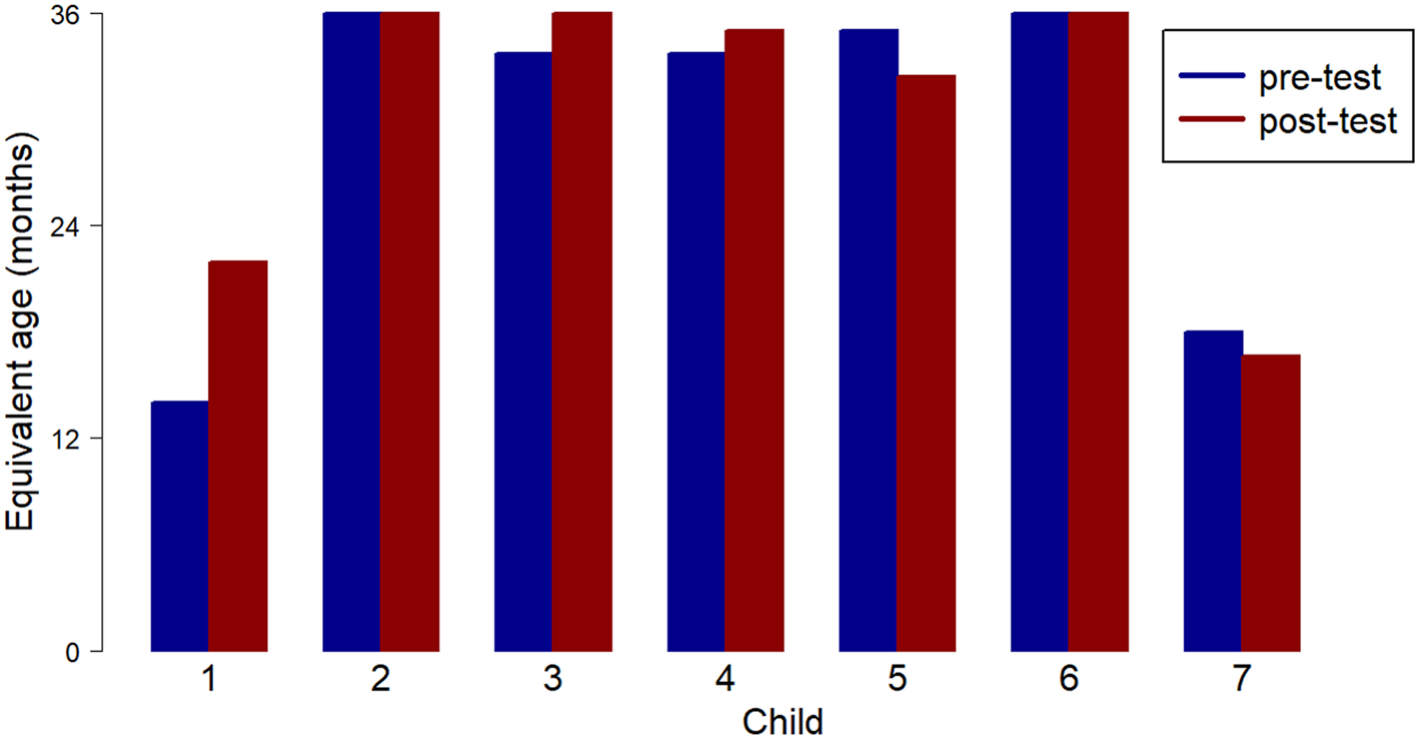
SPT pre and post scores

The pre- and post- approach age equivalent scores for each child on the SPT are shown in Table 2 and graphically in Fig. 1.

**Table 2:** Pre- and post-intervention SPT scores for each participant

Participant	Age (at start of support sessions)	LD / EA	SPT (pre) Age equivalent score	SPT (post) Age equivalent score	Change
1	72 m		14 m	21.9 m	+ 7.9 m
2	71 m		36 + m	36 + m	n/a ceiling
3	79 m		33.7 m	36 m	+ 2.3 m
4	80 m		33.7 m	35 m	+ 1.3 m
5	74 m	EAL	35 m	32.4 m	− 2.6 m
6	63 m	LD + EAL	36+m	36+m	n/a ceiling
7	91 m	LD	18 m	16.6 m	− 1.4 m

It can be seen that for most children pre- and post-approach scores on the SPT were near the uppermost score for this test (36 months), and showed little movement, which could be due to the ceiling effect. Participant 1, for whom the pre-test score was lowest, showed the greatest gain of 8 months on this assessment. Participants 2 and 6 (LD + EAL), scored at ceiling on pre-approach testing. One child with EAL and one child with LD showed a slightly reduced score in the post-test.

**Fig. 2:**
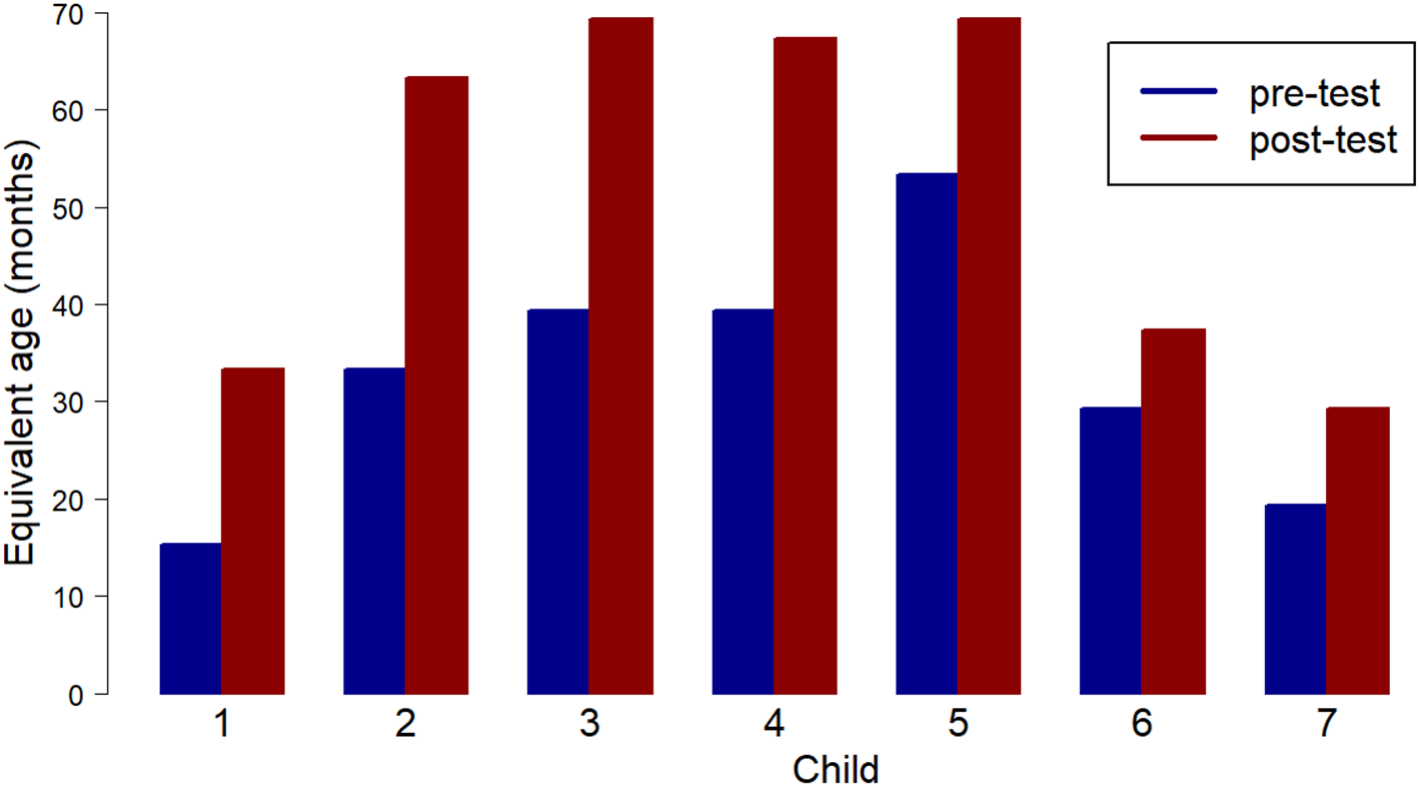
ToPP pre and post scores

The pre- and post- approach age equivalent scores on the ToPP for each child are shown in Table 3, and depicted graphically in Fig. 2.

**Table 3:** Pre-and post-intervention ToPP scores for each participant

Participant	Age	LD/EA	ToPP (pre)	ToPP (post)	Change
1	72 m		15.3 m	33.3 m	+ 18 m
2	71 m		33.3 m	63.3 m	+ 30 m
3	79 m		39.3 m	69.3 m	+ 30 m
4	80 m		39.3 m	67.3 m	+ 28 m
5	74 m	EAL	53.3 m	69.3 m	+ 16 m
6	63 m	LD + EAL	29.3 m	37.3 m	+ 8 m
7	91 m	LD	19.3 m	29.3 m	+ 10 m

The age equivalent scores presented in Table 3 all end with the same decimal point figure of ‘.3’ This is in accordance with the scoring in the ToPP manual (p.36). The ceiling score for the ToPP is 77.3 months

Increases were found in every child’s age-equivalent ToPP score, with increases ranging from + 8 to + 30 months across the participant group. Three children showed increases in their scores of 28 months or more. The ceiling score for the ToPP is 77.3 months.

In the ToPP assessment the greatest age equivalent gains occurred for participants ‘Child 2’, ‘Child 3’ and ‘Child 4’. These participants all completed 10 sessions, all had English as their first language, and all did not have additional learning difficulties. The post-scores for these children were noticeably closer to their chronological age of 6–7 years rather than the pre-score age equivalent level of 3 or 4 years. Child 5, who had English as an additional language, also reached an equivalently high post-score. This child showed a considerably higher pre-score compared to the other children, and consequently the change reflected in the post-score was less than for children 2, 3, and 4. The two children whose scores increased less, child 6 and child 7, had a learning disability, and in one case also had English as an Additional Language.

Child 1 and Child 7 each showed a similar age equivalent pre-score on their ToPP assessment as on their SPT assessment, however, the post score for each child on the ToPP was considerably higher than on the SPT.

Within the ToPP, the overall score for a participant is the total of the scores from 4 separate sections of the test. Section 1 is a very simple play situation of ‘self with everyday objects’, which are a bowl and spoon. Imaginative play is encouraged, however, maximum points can be achieved in this section by imitating the adult. Of the 7 participants, 5 were able to achieve the maximum score at the pre-intervention assessment. There was little change demonstrated in this section

**Fig. 3:**
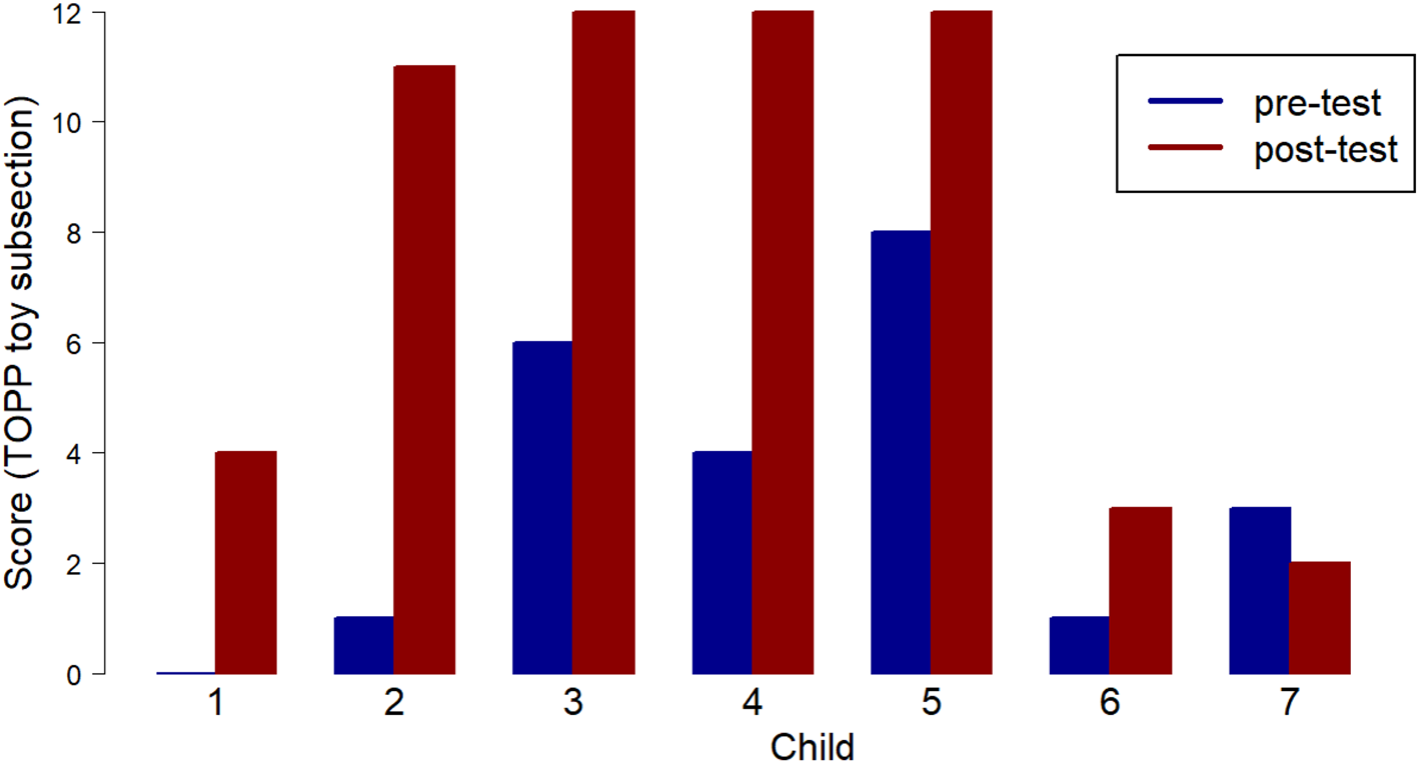
ToPP section 3 scores, representational toy alone

**Fig. 4:**
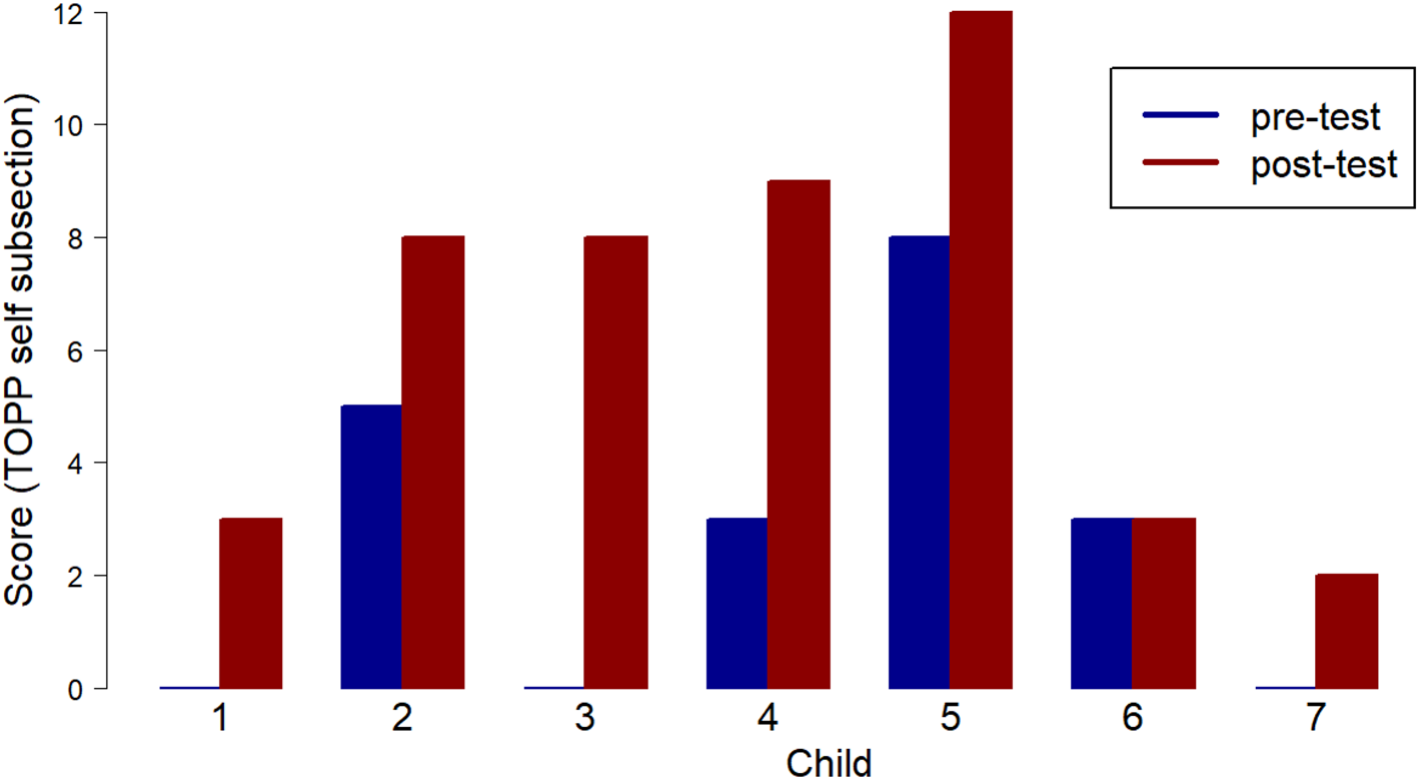
ToPP section 4 scores, self alone

For sections 2, 3 and 4 in the test, only half of the maximum scores can be achieved through imitation, with the rest of the score being gained through generation of new pretence ideas following an elicitation (such as ‘what else could teddy do?). It is in sections 3 and 4, where the child is required to reference non-present objects, attribute emotions, reference substitutions, and act out scenarios involving only the representational toy (teddy) or themselves (e.g. ‘show me how you can be a rabbit.’), that the development of novel generated imaginative play is demonstrated for many of the cohort (Figs. 3 and 4).

Figure 3 shows that three of the participants achieved the maximum score on section 3, following the approach sessions, with one further participant scoring very close to maximum.

Child 1 had not demonstrated pretend play with the representational other in this section at the pre-test, showing no interest in playing with the teddy, but scored 4 in the post-test being able to copy teddy driving a car, demonstrate teddy having a drink, and when asked ‘what else can teddy be?’ holding the teddy high and saying ‘a moon’. The representational other used in the ToPP assessment (a relatively small teddy), had not been used in any of the Playboxes sessions where the representational others used were a wide variety of soft toys and puppets.

As can be seen in Fig. 4, in section 4 (Self Alone) 6 of the participants demonstrated increased scores in imaginative play in relation to themselves alone.

Scores above 6 could only be achieved where new imaginative ideas had been generated by the child. One child, ‘Child 5’, with EAL, increased their score to the maximum in section 4. Child 1, Child 3 and Child 7 had not demonstrated pretend play in this section before the Playboxes sessions.

Although ‘Child 5’ and ‘Child 6’ completed fewer sessions (8 and 7 respectively), increased scores were demonstrated for these participants. No link was observed between the increase in age equivalent gains for the participants and the profession of the adult play partner. Similarly, the two children who had the same professional play partner showed different individual scoring patterns. Additionally, no pattern was observed in the outcomes for the children in relation to whether or not the professional administering the ToPP was the same professional who was implementing the Playboxes sessions.

## Discussion

Post-intervention ToPP scores showed increases in pretend play for all of the children in the study, including those with an EAL or LD. For some children these increases reflected an increase of over 2 years in age equivalent scores. It is notable that the increase in age equivalent months shown by the participants on the ToPP scores considerably exceeded the length of time in which the participants were involved in the Playboxes sessions. This would indicate that the changes in the scores cannot be explained by natural maturation and development of the participants over time.

The most substantial increases in post-intervention ToPP scores were found for those children in the study who did not have additional learning difficulties and who attended 10 sessions. The increases in the age equivalent scores for this group of children ranged from + 18 months to + 30 months. Nevertheless individual differences are clearly apparent for each of the children in relation to both initial pre-scores and increased post-scores, reflecting very individual patterns of change which would be expected in a study of autistic children (Magiati et al., 2007). The two children whose age equivalent score increases on the ToPP were smaller, had an additional learning difficulty, and in one case also had English as an additional language. Previous research has similarly reported increases in the ToPP scores of children with additional learning difficulties following play programmes with trained adults or peers. In a study using a structured teaching intervention, Sherratt (2002) reported increases in post intervention ToPP scores ranging from 2–12 months, for four autistic participants with additional learning difficulties. Although showing generally a little less progress than the two autistic children with learning difficulties in this current study, who showed increases of + 8 months and + 10 months, the increased scores found in Sherratt’s study are within a similar range of development.

Scores on the SPT also showed individual differences. For many of the participants, including a participant with LD and EAL, the scores were near to, or at, the ceiling score, which while demonstrating functional symbolic pretence abilities for these children, however, provided only limited age-related information on this due to the constraints of the assessment test and the ages of the participants. Nevertheless, for two of the children, Child 1 and Child 7, low age equivalent scores in relation to functional symbolic play were revealed by the test. Both children also scored similarly on their ToPP pre-test and on their SPT pre-test. It is notable, however, that the post score for each child on their ToPP was considerably higher than on the SPT. This may reflect the somewhat greater interaction with the adult in the ToPP compared to the SPT and the greater imitative opportunities of pretend play that this supports. It could also reflect the greater opportunities within the ToPP to demonstrate novel symbolic play ideas such as object substitution and reference to imagined properties in relation to persons and objects which have been directly supported within the interactive pretend scenarios within Playboxes.

The increases in ToPP score were seen, for many of the children, to arise from developments in the generation by the children of new imaginative pretence and novel construction of imaginative ideas. The developments in pretend play over a period of 10 weeks seen in the children in the study resonates with the theoretical position that the lack of pretend play often reported in autistic children can be argued to reflect, not an alteration in imaginative processes underpinning pretence, but differences in being drawn to engage in playful pretence interactions and to generate pretend play ideas, (Hobson et al., 2009; Jarrold, 1993; Kasari et al., 2013). This difference in motivational draw could affect uptake of opportunities to engage in the joint creation of pretence, in sharing perspectives, and learning about pretence from others. Results in this study would support the theoretical view that play processes can be supported through facilitative joint play which motivates and supports the child to engage interpersonally (Hobson et al., 2009).

The increases in the ToPP scores could be argued to reflect the particular qualities of the Playboxes method, which encourages joint engagement and the co-creation of meanings, within a playful setting with highly positive shared affect. Playboxes aims to be fun and motivating, providing opportunities to imitate and to lead, to build shared expectations and anticipations and shared interpersonal effectiveness. The representative other is included as an active participant in various imaginary scenarios enabling observation and demonstration of emotions and actions in relation to people and interpersonal interactions, which can be argued to help develop conceptual understanding of personhood as well as understanding of ‘pretence’ itself. It is this type of involved playful engagement which has been identified in previous research works as encompassing the qualities of interaction necessary to facilitate symbolic pretend play (Hobson et al., 2009; Sherratt, 2002) and Hobson et al (2013) highlight the role of communication and social interaction underpinning pretend play. The specific focus of the engagement between the child and the play partner within the Playboxes approach, created by their own intersubjective understandings as the sessions progress, enables individual differences in the children to be supported. In this way Playboxes works with the individual child and is tailored to the child. Nevertheless, Hobson et al. (2013) report that the pretend play of autistic children is not always ‘playful’ pretence, and Kasari et al. (2013) emphasise the need to examine the affective experience of play for autistic children. Playfulness in pretence was not a component specifically analysed in this research and incorporation of a measure of playfulness in pretend play in future research could deepen understanding of processes supporting the use of pretence in autistic children.

The standardised assessments measured pretend play abilities in the child in a setting separate from the familiar context of the Playboxes interaction, and where the role of the adult is limited in relation to co-construction of pretence. Potential practice effects which may have been developed by the child and adult play partner within the Playboxes sessions were avoided as part of the assessment, and it was the pretend play generated by the child using unfamiliar toys in a structured and prescribed sequence of activities in an unfamiliar context which was examined. The use of this assessment enabled the observation of pretend abilities in the children expressed without being in a fully interactive and facilitative context, thereby supporting the position that the increases in pretend play generated by the children demonstrate the generalization of the pretend play abilities for the child. Although the role of the adult is limited and prescribed in the ToPP assessment, there can be some prompting and eliciting involved, which research has shown can in itself support the generation of play for autistic children (Charman & Baron-Cohen, 1997; Jarrold et al., 1993, 1996), however, any such influence would apply to both pre and post scores.

The results also indicate that the Playboxes method can be effectively incorporated into ongoing practice. This is valuable as it means that professionals can use the method at the point of concern, and for as long as the approach is considered to be useful. Intervention studies are often time-limited, and the sustainability of impact not able to be established (Kasari et al., 2013). An intervention to support interpersonal engagement, joint attention, and pretend play abilities, which can be embedded into ongoing professional practice, would enable support for these key abilities to be accessible, widely deliverable, and sustained. The ‘Playboxes’ joint-play approach is particularly suitable for incorporation into ongoing practice because of its flexibility in setting, weekly delivery in short sessions, and low amount of resources needed. The diversity of the sample group in this study indicates that the approach can be effectively used with children with a range of different needs and in their school setting, highlighting the applicability of the approach. The study also demonstrates that a range of professionals can successfully deliver Playboxes. The accessibility of the approach would make it suitable for not only trained professionals but also potentially for parents to use.

While showing positive results, this small scale pragmatic study has a range of limitations. In using the approach as part of ongoing practice, the study involved a relatively small sample of non-randomly selected participants. Individual patterns of change and low numbers mean that results must be interpreted with caution. The considerations of embedding the approach in practice also resulted in pragmatic decisions in relation to the study design, and precluded the use of a control group comparison. The fact that pre- and post- assessments were not in all cases implemented by a different professional to the one who was involved in the Playboxes sessions with the child further reduced the level of control in the study. Child and professional absence also affected the number of sessions carried out, with some children participating in slightly fewer sessions than the others, although this alone did not appear to lead to smaller change in age-equivalent score. Limitations to studies using repeated assessments can be that increased scores could reflect practice effects on the assessment measure itself, but the type of developments seen on the ToPP, such as moving from no play at all with the representational other to both imitative and novel pretence, suggest these are not practice effects arising from the exposure to ToPP itself.

## Conclusion

The evidence from this small-scale study using independent standardized measures of pre and post change indicates the effectiveness of the Playboxes approach in supporting the development and use of pretend play abilities in autistic children, and that these abilities can be supported. All participants demonstrated increased pretend play, including participants with LD and EAL. It is argued that the applicability of Playboxes for all children reflects the individualized nature of the co-created shared play experiences within the Playboxes sessions. The playboxes method was shown to be suitable for inclusion in ongoing practice for a range of professionals, making it an accessible approach which could be widely used. The use of independent standardised measures to assess the effectiveness of the approach in supporting developments in pretend play indicated that developments in pretence abilities from Playboxes were generalised to an unfamiliar context. Increasing play abilities places children in a position where social interaction and joint play can be enhanced, which can be argued to impact on friendships, and also on cognitive and linguistic development for a child. Pretend play abilities can be understood to aid educational inclusion and social well-being, and use of this approach in practice could be of value for autistic children.
